# Stomata Are Driving the Direction of CO_2_-Induced Water-Use Efficiency Gain in Selected Tropical Trees in Fiji

**DOI:** 10.3390/biology13090733

**Published:** 2024-09-19

**Authors:** Wuu Kuang Soh, Charilaos Yiotis, Michelle Murray, Sarah Pene, Alivereti Naikatini, Johan A. Dornschneider-Elkink, Joseph D. White, Marika Tuiwawa, Jennifer C. McElwain

**Affiliations:** 1 National Botanic Gardens (OPW), Glasnevin, D09 VY63 Dublin, Ireland; 2 Department of Biological Applications and Technology, University of Ioannina, 45110 Ioannina, Greece; chyiotis@uoi.gr; 3 Department of Botany, School of Natural Sciences, Trinity College Dublin, College Green, Dublin 2, D02 PN40 Dublin, Ireland; mnmurray40@gmail.com (M.M.); jmcelwai@tcd.ie (J.C.M.); 4 School of Geography, Earth Science and Environment, University of the South Pacific, Laucala Campus, Suva 679, Fiji; sarah.pene@usp.ac.fj; 5 Forest Research Division, Colo-i-Suva Station, Ministry of Forestry, Suva 679, Fiji; drdnaikatini@gmail.com; 6 School of Politics and International Relations, University College Dublin, Belfield, D04 V1W8 Dublin, Ireland; jos.elkink@ucd.ie; 7 Department of Biology, Baylor University, Waco, TX 76798, USA; joseph_d_white@baylor.edu; 8 South Pacific Regional Herbarium, University of the South Pacific, Laucala Campus, Suva 679, Fiji; marika.tuiwawa@usp.ac.fj

**Keywords:** intrinsic water-use efficiency, stomatal conductance, photosynthesis, carbon dioxide, herbarium, intraspecific trait variation, tropical trees, Fiji

## Abstract

**Simple Summary:**

Understanding how plants respond to increasing atmospheric CO_2_ is crucial for predicting future climate interactions. However, the long-term effects of rising CO_2_ on plant physiology, especially in tropical regions, are not well known. To investigate this, we studied how a CO_2_ increase of about 95 ppm from 1927 to 2015 affected five tropical tree species in Fiji. We analysed historical leaf samples to measure the following two key traits: how efficiently the trees use water (intrinsic water-use efficiency) and the maximum rate of conductance through leaf pores (maximum stomatal conductance). Our results showed that the responses to rising CO_2_ varied significantly by species. Generally, the number of stomata on the leaves was more important than their size in determining the trees’ response to higher CO_2_ levels. While photosynthesis is a major factor in improving the water-use efficiency, changes in stomatal conductance primarily drive this trend across different species. Trees that showed greater increases in the water-use efficiency also displayed a greater reduction in stomatal conductance. Overall, our study shows the importance of considering differences in the maximum stomatal conductance when predicting how different tree species will react to increasing CO_2_ levels.

**Abstract:**

Understanding plant physiological response to a rising atmospheric CO_2_ concentration (*c*_a_) is key in predicting Earth system plant–climate feedbacks; however, the effects of long-term rising *c*_a_ on plant gas-exchange characteristics in the tropics are largely unknown. Studying this long-term trend using herbarium records is challenging due to specimen trait variation. We assessed the impact of a *c*_a_ rise of ~95 ppm (1927–2015) on the intrinsic water-use efficiency (iWUE) and maximum stomatal conductance (*g*_smax_) of five tropical tree species in Fiji using the isotopic composition and stomatal traits of herbarium leaves. Empirical results were compared with simulated values using models that uniquely incorporated the variation in the empirical *g*_smax_ responses and species-specific parameterisation. The magnitude of the empirical iWUE and *g*_smax_ response was species-specific, ranging from strong to negligible. Stomatal density was more influential than the pore size in determining the *g*_smax_ response to c_a_. While our simulation results indicated that photosynthesis is the main factor contributing to the iWUE gain, stomata were driving the iWUE trend across the tree species. Generally, a stronger increase in the iWUE was accompanied by a stronger decline in stomatal response. This study demonstrates that the incorporation of variation in the *g*_smax_ in simulations is necessary for assessing an individual species’ iWUE response to changing *c*_a_.

## 1. Introduction

Land plants play an important role in regulating Earth’s hydrological cycle and energy balance by transpiring water through the stomata on their leaves. While the primary response of plants to an increasing atmospheric CO_2_ concentration (*c*_a_) is to increase the assimilation rates (*A*), it is often accompanied by reducing diffusive stomatal conductance (*g*_s_) in order to minimise transpirational water loss [1]. This results in an increase in the plant intrinsic water-use efficiency, which is a ratio of the carbon gain to water loss under the same evaporative demand (iWUE = *A*/*g*_s_) [2]. The historical iWUE can be estimated from the stable carbon isotope (δ^13^C) in plant tissue on the basis of the relationship between carbon isotope discrimination and the iWUE (see Section 2). One method of studying long-term and iWUE responses to *c*_a_ in plants involves using the δ^13^C extracted from herbarium leaves or wood tissue in tree rings. Evidence from herbarium leaf and tree-ring archives demonstrated that the iWUE of C3 plants has increased in proportion to the rise in *c*_a_ over the past few decades in both the tropical [3] and temperate [4] regions.

Tropical forests are dominant contributors of water vapour to the atmosphere via transpiration, comprising approximately 35% of global precipitation, with estimates as high as 70% for the tropics [5]. Changes in the gas-exchange capacity of these forests would likely impact Earth system processes, including planetary albedo [6], surface runoff [7], and biomass accumulation [8]. Tropical forests also play an important role in the global carbon cycle, since they store approximately half of the carbon in the terrestrial biosphere and account for about a third of global terrestrial productivity [9]. However, despite their importance to the global soil–plant–atmosphere continuum, studies on physiological response, such as the changes in the *g*_s_ and iWUE, associated with an increasing *c*_a_ in tropical tree species, are very much limited [4,10]. Addressing this gap is important because ecosystems in the tropics differ from the temperate areas in climate, soil, community assemblage, and ecological attributes, which are expected to influence how species respond to rising *c*_a_ [10].

Evidence from herbarium leaf samples shows that the long-term increase in *c*_a_ has contributed to changes in the stomatal micromorphology through the reduction in stomatal density (*SD*) and the maximum pore size (*a*_max_) [11,12,13]. Stomatal traits are key in determining the micromorphological limit of the theoretical maximum stomatal conductance (*g*_smax_) [14]. It is widely known that, because stomata respond dynamically to the environment, through the turgor pressure-mediated closure of the stomatal pore, *g*_smax_ is rarely observed in field conditions [15,16]. Nonetheless, *g*_smax_ is tightly correlated with *g*_s_ [14,15,17,18], and therefore can be used as a proxy in studying long-term historical changes in stomatal conductance [12].

One common issue with using herbarium samples to track temporal changes in the iWUE and stomatal traits is the high variability in the data due to sampling gaps and intraspecific trait variation. A proposed solution to minimise the variability due to sampling gaps is to sample at a high temporal resolution [12,19]. However, this is often difficult in practice due to the sparsity of the geographical and temporal scale of herbarium collections, an issue which is much more prevalent in the tropical areas due to low collection efforts [20]. Intraspecific variability in plant traits is the result of heritable genetic and epigenetic differences, and plastic responses to biotic and abiotic conditions [21]. For example, the collection of plant samples for an herbarium from different growing environments, periods, and populations can contribute significantly to intraspecific variability in traits.

The key research objectives of this study are first to quantify how tropical tree species have changed their iWUE and *g*_smax_ in response to the long-term anthropogenic rise in *c*_a_ in the Southwest Pacific region of Fiji. The second objective is to investigate the influence of stomata (*g*_smax_ and *g*_s_) on the iWUE over the rising c_a_. In the preceding analysis, we considered the intraspecific trait variation observed in herbarium specimens. For this study, the following five evergreen broadleaved species from Fiji were selected: *Amaroria soulameoides* A. Gray, *Astronidium confertiflorum* Markgr., *Dillenia biflora* (A.Gray) Martelli ex Guill., *Elattostachys falcata* (A.Gray) Radlk., and *Gnetum gnemon* L. Herbarium leaf samples collected between 1927 and 2015 were utilised to infer the long-term iWUE and *g*_smax_ from δ^13^C and stomatal traits, respectively. We simulated the iWUE, *A*, and *g*_s_ response to *c*_a_ by using the following two independent models: (a) a biogeochemical model (BiomeBGC) with a statistical procedure and (b) a simple mechanistic empirical–biochemical model—both of the models incorporate the intraspecific variation in *g*_smax_ and apply species-specific parameterisation from leaf functional traits. We also assessed the phenotypic space of stomatal traits by comparing the results of stomatal morphologies with data from subtropical tree species in Florida.

## 2. Materials and Methods

### 2.1. Study Area, Species, and Herbarium Samples

Fiji is situated in the South Pacific region, with a total land area of 18,376 km^2^, of which 85% consists of the two largest islands, Vanua Levu and Viti Levu. The country has a warm tropical climate with a sea-level temperature averaging to about 22 °C in July and 26 °C in January. Annual rainfall on the main islands is between 2000 mm and 3000 mm on the coast and low-lying areas [22]. The following five common tree species in Fiji were selected for this study: *Amaroria soulameoides*, *Dillenia biflora*, *Astronidium confertiflorum*, *Elattostachys falcata*, and *Gnetum gnemon*. Four of them, *A. soulameoides*, *A. confertiflorum, D. biflora*, and *E. falcata*, are angiosperm species occurring in both open and closed forests. *Gnetum gnemon* is the only gymnosperm species in this study, and it is frequently found in closed forests. Two of the species, *A. soulameoides* and *A. confertiflorum*, are endemic to Fiji, while *D. biflora* and *E. falcata* are confined to Fiji and the Southwest Pacific islands. *Gnetum gnemon* is widespread in Asia and the Pacific region.

Herbarium leaf samples of the five species collected between 1927 and 2015 from the two adjacent main islands in Fiji, Vanua Levu and Viti Levu, were used in this study. The leaf samples used here were mainly from the South Pacific Regional Herbarium (SPRH), collected between 1927 and 1996, and some from Trinity College Dublin Herbarium (TCD), collected in 2015 by the same team in this study [23], as well as the CLAMP (Climate-Leaf Analysis Multivariate Programme) herbarium deposited in the Smithsonian Institute (US), collected by Wolfe in 1988 [24] (see the Supplementary Materials dataset for the origin of the samples). A total of 246 herbarium leaf samples from five species were used in this study. To minimise the effect of light on the variation in the δ^13^C and stomatal traits, only leaves collected from the outer canopy were used. This was achieved by sampling leaves from flowering or fruiting herbarium specimens in the SPRH collections. The specimens collected in the 2015 fieldwork and the CLAMP herbarium that were used in this study were all known to be collected from the outer canopy [23,24]. The herbarium specimens were collected from within arm’s reach, and if they were out of reach, a pole no taller than 5 m was used to collect them. The historical specimens at the SPRH herbarium were also likely to be collected in the same manner (Tuiwawa, M., personal communication, May 2015). The foregoing protocol ensured uniformity in the height of the canopy from which the specimens were collected. Only leaves that were free (visually) from fungus infestation and insect herbivory were used in this study. To minimise the effect of altitudinal CO_2_ partial pressure, only specimens collected below 600 m a.s.l. were used. Collection years were recorded from the herbarium labels.

### 2.2. Analysis of Leaf Stomatal Traits

An approximately 2 cm^2^ nail varnish imprint was obtained directly from abaxial leaf surfaces (negatives) at the middle region of the blade from each leaf (one leaf per herbarium sample). On each cuticle impression, the images of five fields of view were randomly taken at 200× magnification using a Leica DM2500 microscope with a Leica SDC300FX camera (Leica Microsystems, Wetzlar, Germany) and Syncroscopy Automontage (Syncroscopy, Cambridge, Cambridgeshire, UK) digital imaging software. Stomatal density was counted on a 0.09 mm^2^ square frame using ImageJ2 software [25] following the protocol from Poole and Kürscher [26]. Five counts of stomatal density (*D*) per leaf were averaged, and this value was used for the statistical analysis and calculation of the theoretical maximum stomatal conductance (*g*_smax_). One field of view per leaf impression was photographed at 400x magnification. In each of these 400x images, the pore length and guard cell width of ten randomly selected stomata were measured (Supplementary Data Figure S1). For each cuticle impression, the maximum pore size (*a*_max_) was calculated from each of the stomatal pore length measurements and, subsequently, ten *a*_max_ values were averaged for use in the statistical analysis and calculation of the *g*_smax_. The theoretical maximum stomatal conductance, *g*_smax_, was calculated using the following diffusion equation [27,28]:

(1) $$g_{\mathrm{smax}}=\frac{\frac{d_{w}}{v}\cdot D\cdot a_{\max}}{p+\frac{\pi }{2}\;\sqrt{\frac{a_{\max}}{\pi }}}$$

where *d*_w_ = the diffusivity of water vapour at the standard reference of 25 °C (2.49 × 10^−5^ m^2^ s^−1^) and *v* = the molar volume of the air (2.24 × 10^−2^ m^3^ mol^−1^), which are both constants; *D* is the stomatal density (m^−2^); *a*_max_ is the maximum pore area (m^2^), calculated as an ellipse using the stomatal pore length, *L* (m), = π*L*^2^/8; *p* is stomatal pore depth (m), which is considered to be equivalent to the width of a guard cell, assuming that guard cells are inflated to a circular cross-section [28].

### 2.3. Analysis of Leaf Functional Traits

One-half of each herbarium leaf blade was used for the leaf mass per area (LMA) analysis, and the other half was used for the stable carbon isotope (δ^13^C) and leaf nitrogen per area (N_area_) elemental analyses. The LMA was determined by dividing the dry leaf weight by the rehydrated leaf area. The specific leaf area (SLA) was calculated as the inverse of the LMA. The leaf area shrinkage from drying can be reversed by rehydration [29]. For the δ^13^C, total nitrogen, and carbon elemental analyses, dried leaf fragments were placed with a tungsten bead in Eppendorf tubes and finely ground in a mixer mill (Tissue Lyser, Qiagen Inc., Valencia, CA, USA). Each sample (~3 mg) was then enclosed in a tin capsule using a crimper plate. The samples were analysed for δ^13^C, C, and N using a PDZ Europa ANCA-GSL elemental analyser interfaced with a PDZ Europa 20–20 isotope ratio mass spectrometer (Sercon Ltd., Cheshire, UK) at the UC Davis Stable Isotope Facility, University of California, Davis, CA, USA. The instrumental error was ±0.18‰ (per mil) for ^13^C (standard deviation). The carbon isotope composition was calculated as follows:

(2) $$\delta^{13}C\;\left(‰\right)=\frac{\left(R_{\mathrm{sample}}-{\;R}_{\mathrm{standard}}\right)}{R_{\mathrm{standard}}}\;$$

where R_sample_ and R_standard_ are the ^13^C/^12^C ratio of the sample and the international standards Vienna Pee Dee Belemnite, respectively. The carbon isotopic discrimination (∆_plant_) is given as follows [30,31]:

(3) $$\Delta_{\mathrm{plant}\;}=\frac{\left(\delta^{13}C_{\mathrm{air}}-{\;\delta }^{13}C_{\mathrm{plant}}\right)}{1+\left(\delta^{13}C_{\mathrm{plant}}/1000\right)}$$

In relation to the intercellular CO_2_ (*c_i_*) and ambient CO_2_ concentration (*c_a_*), the ∆_plant_ in the C3 leaves is given as follows [30,31]:

(4) $$\Delta_{\mathrm{plant}\;}=a+\left(b-a\right)\left(c_{i}/c_{a}\right)$$

where *a* is the fractionation due to the diffusion in air (4.4‰), and *b* is an empirically derived parameter representing the combined fractionations that occur during and after photosynthesis in a leaf (27‰) [32]. Equation (4) is widely used, and assumes that the effects of the boundary layer, internal conductance, photorespiration, day respiration, and resource allocation are negligible. More comprehensive models of ∆_plant_ include several elements, such as photorespiration, day respiration, and the CO_2_ mole fractions in the ambient air, at the leaf surface, in the intercellular air spaces, and at the chloroplast (*c*_c_), and are often difficult to parametrise [30,33]. Since the aim of our analysis was only to calculate an integrated *c*_i_/*c*_a_ ratio from dried leaf matter, the simplified model of Equation (4) has been repeatedly shown to be highly accurate and a reasonable compromise when an appropriate *b* (i.e., 27‰ in the case of leaf bulk material) is used [32].

The historical annual δ^13^C_air_ and *c*_a_ between 1927 and 2015 were obtained from published data [34,35,36]. The intrinsic water-use efficiency (iWUE) can be expressed as the ratio of photosynthesis (*A*) and leaf conductance to water vapour transfer (g_s_) in Equation (5) below [31] using the *c*_i_/*c*_a_ calculated from Equation (4) and *c_a_*, as follows:

(5) $$\mathrm{iWUE}=A/g_{s}=c_{a}\;\left(1\;-\;c_{i}/c_{a}\right)/1.6=c_{a}\left(1\;-\;\left(\Delta \;-\;a\right)/\left(b\;-\;a\right)\right)/1.6$$

The intrinsic water-use efficiency inferred from the δ^13^C is an average estimate of the iWUE over a leaf lifespan, i.e., time-integrated over the lifetime of the leaf.

### 2.4. Phenotypic Space of Stomatal Density (D) and Maximum Stomatal Pore Area (a_max_) in Plant Communities

We are interested in understanding how the *g*_smax_ of individual species and plant communities are constrained by the variability of *a*_max_ and *D* in their phenotypic space. From a morphological standpoint, the range of *g*_smax_ values is delimited by the constraints imposed by the limits of stomatal traits, specifically *a*_max_ and *D* (Equation (1)). The boundaries set by *a*_max_ and *D* values reflect the phenotypic space in stomatal traits. Therefore, the scope of response of *g*_smax_ to rising *c*_a_ is also confined by the phenotypic space of stomatal traits.

Here, we evaluate the phenotypic space of stomatal traits occupied by the plant community in Fiji situated in the tropical biome and compare it with the phenotypic space observed in the plant community of a subtropical biome in Florida. Given the limited research available on the stomatal traits of tropical trees, we drew upon data from published historical stomatal trait data, *D* and *a*_max_, from eight tree species in Florida (from the years 1927 to 2009) [37] The eight tree species include *Acer rubrum* L., *Ilex cassine* L., *Morella cerifera* (L.) Small, *Pinus elliottii* Engelm., *Pinus taeda* L., *Quercus laurifolia* Michx., *Quercus nigra* L., and *Taxodium distichum* (L.) Rich.

To quantify the probability density overlap between the plant communities in Fiji and Florida in a contour plot, the point pattern of the specimens in the 2-dimensional space as defined by the log of *a*_max_ and the log of *D* is first converted into a smoothened density over a grid of 100 by 100 cells using a 2-dimensional kernel density estimation based on the built-in kde2d function in the MASS library in R [38]. Based on the estimated probability densities of stomatal traits for Fiji and Florida, the Jensen–Shannon divergence (JSD) [39] was calculated assuming equal weighting for the two plant communities, as follows:

(6) $$\mathrm{JSD}=\frac{1}{2}\left(\sum \left(p\;\log\left(\frac{2p}{p+q}\right)\right)+\sum \left(q\log\left(\frac{2q}{p+q}\right)\right)\right)$$

where p and q represent the two probability distributions of the stomatal traits of the plant community in Fiji and Florida, respectively. The Jensen–Shannon divergence is a divergence measure bounded between 0 and 1, representing complete overlap and complete divergence, respectively.

### 2.5. Meteorological Data

Near-surface, daily meteorological data required for the BiomeBGC simulations included the maximum, minimum, and mean temperature, precipitation, vapour pressure difference, global radiation, daylength, and annual *c_a_*. Daily records of precipitation and temperature (minimum and maximum) were taken from the CPC Global Daily Gridded (0.5° × 0.5°) meteorological data covering the period from 1979 to 2015 [40,41]. To our knowledge, these are the only available source of daily meteorological data that include Fiji. The R statistical software (R version 4.4.1) [42] and R package *ncdf4* [43] were used to extract the daily Fiji meteorological data from the complete CPC Global Daily Gridded dataset from the year 1979 to 2015 at a latitude between 16°15′ S and 18°15′ S, and a longitude between 177°45′ E and 179°15′ E. Next, using the meteorological data as the input, the microclimate simulator MTCLIM [44,45] was used to estimate the daily downward shortwave radiation, vapour pressure difference, and daylength. Meteorological parameters from all of the grids were then averaged. The historical annual *c*_a_ between 1927 and 2015 was obtained from published data [34,36]. The Mean Annual Precipitation (MAP) and annual self-calibrating Palmer Drought Severity Index (scPDSI) between 1927 and 2015 were obtained from the 0.5° × 0.5° resolution Climate Research Unit data (CRUTS v.4.0) [46] gridded dataset.

### 2.6. Climatic Conditions in Fiji (1927–2015)

An increase in *c*_a_ of ~95 ppm (from ~305 ppm to ~400 ppm) between 1927 and 2015 in Fiji was accompanied by an increase in the Mean Annual Temperature (MAT) of 0.18 °C, from an average 23.84 °C in the first three decades to an average 24.02 °C in the last three decades (Supplementary Data Figure S2a–b). There were extreme interannual fluctuations in the MAT and MAP since the 1970s, indicating a clear trend of drought and tropical cyclones that correspond to El Niño events [47,48,49] (Supplementary Data Figure S2b–d). The El Niño Southern Oscillation is known to strongly affect climatic variability in the Pacific and is connected to the occurrence of extreme weather such as drought and tropical cyclones [50]. During this period, three record-strong El Niño events were documented in 1982–1983, 1997–1998, and 2015–2016 [51]. To check the effects of these climatic conditions on the iWUE, we performed a bivariate regression between the iWUE and scPDSI, and a multiple regression between the iWUE and two covariates, *c*_a_ and MAP.

### 2.7. Modelling Intrinsic Water-Use Efficiency Response (ΔiWUE/Δc_a_), Stomatal Conductance Response (Δg_s_/Δc_a_), and Photosynthesis Response (ΔA/Δc_a_) to Rising c_a_, and Incorporating Intraspecific Variability of g_smax_

Our general approach was to include the uncertainty of empirical *g*_smax_ responses, which is largely a reflection of the intraspecific variation and, to some extent, data gaps in the simulations of physiological responses (ΔiWUE/Δ*c*_a_ and Δ*g*_s_/Δ*c*_a_). The advantage of this approach is that the confidence intervals (CIs) derived from the intraspecific variation in *g*_smax_ can be placed on the modelled iWUE and *g*_s_. Additionally, all of the simulations were run on species-specific parameterisations. The following describes the two modelling methods used in this study.

#### 2.7.1. BiomeBGC Model (BiomeBGC*) Simulation

BiomeBGC version 4.2 [52] (available online at http://www.ntsg.umt.edu, accessed on 20 January 2017) was used in this study. BiomeBGC is a biogeochemical model that simulates the storage and fluxes of water, carbon, and nitrogen in a terrestrial ecosystem [53,54]. Commonly, the BiomeBGC model simulation requires daily meteorological data, annual *c*_a_, general environment information, and species-specific parameters describing the ecophysiological characteristics of the plant (e.g., one value of *g*_smax_ for each species). However, in this study, a statistical procedure was included to generate a sample of *g*_smax_ values (see the description below).

Daily meteorological data from 1979 to 2015 were used in the simulation, as this is the earliest available daily data for Fiji (Supplementary Data Table S1). Instead of using the default values for the evergreen broadleaf forest (EBF) vegetation type as provided by the BiomeBGC model [53], we used species-specific parameterisation based on species-specific N_area_, LMA, the maximum rate of RuBisCO carboxylation at 25 °C (*V*_Cmax,25 °C_), and the fraction of leaf nitrogen in RuBisCO (Supplementary Data Tables S1 and S2). For each species, the *V*_Cmax,25 °C_ values were calculated using the species-specific average LMA and C:N values, following Thornton and Running [55]. We calibrated the fraction of leaf nitrogen in RuBisCO by rerunning the simulations above (using the average *g*_smax_ as the parameter) with different fraction values until the average *A* values were close to the average empirical values, i.e., the fraction value that resulted in the simulated *A* closest to the average empirical *A* was used for the BiomeBGC parameterisation. The average empirical value of *A* for the following five species was taken from a dataset of gas exchange from Soh et al. [4] (See Supplementary Data), including *Amaroria soulameoides* (15 µmol m^−2^ s^−1^), *Astronidium confertiflorum* (13 µmol m^−2^ s^−1^), *Dillenia biflora* (14 µmol m^−2^ s^−1^), *Elattostachys falcata* (9 µmol m^−2^ s^−1^), and *Gnetum gnemon* (9 µmol m^−2^ s^−1^). With the exception of the *g*_smax_ value, each of the parameters above were input as a single constant value in BiomeBGC, which is the standard way of inputting the parameters for this model. In this study, we allowed *g*_smax_ to vary annually instead of being treated as one fixed parameter in the BiomeBGC model, i.e., the simulations were performed by using species-specific *g*_smax_ values for every year (g*_smax,*s,t*_) as the input in the simulation, and thereby this approach takes into account the statistical uncertainty of the empirical *g*_smax_ (Supplementary Data Figure S3). This statistical uncertainty is the result of the intraspecific trait variation in the empirical *g*_smax_ and data gaps. To generate the g*_smax*,s,t*_ values, a set of 10,000 coefficients values, i.e., intercepts and slopes (β*_s_), were sampled from the multivariate normal sampling distribution [56] from the regression of the empirical *g*_smax_ and *c*_a_, i.e., *g*_smax,*s,t*_ = *β*_1s_ + *β*_2s_[CO_2_]*_i_* (Supplementary Data Figure S3a–b). This step crucially captures the uncertainty of the empirical *g*_smax_. Next, the species-specific *g*_smax_ values for every year (g*_smax,*s,t*_) were calculated from each of the 10,000 sets of coefficient values (Supplementary Data Figure S3c). Following this, the simulations were executed in BiomeBGC using g*_smax,*s,t*_ and species-specific parameterisation (N_area_, LMA, *V*_Cmax,25 °C_, and the fraction of leaf nitrogen in RuBisCO) to generate daily stomatal conductance (*g*_s*,d*_), daily sunlit canopy components of photosynthesis (*A*_s,d_), and daily iWUE (iWUE_s,d_) values (Supplementary Data Figure S3d). The iWUE_s,d_ values were obtained by calculating the ratio of *A*_s,d_ and *g*_s,d_. Then, the mean annual values of the iWUE, *A*, and *g*_s_ for each species (iWUE*_s,t_*, *A_s,t_*, and *g*_s,*s,t*_) were calculated from the daily values (Supplementary Data Figure S3e). Next, the iWUE responses to the rising *c*_a_ and ΔiWUE/Δ*c*_a_ (*δ*_2*s*_) were obtained by regressing the *iWUE*_s,t_ with c_a_. We obtained Δ*A*/Δ*c*_a_ (*γ*_2*s*_) and Δ*g*_s_/Δ*c*_a_ (*η*_2*s*_) by regressing *A_s,t_* and *g*_s,*s,t*_, respectively, with *c*_a_ (Supplementary Data Figure S3f). On the basis of the 10,000 runs of the otherwise deterministic simulation, a 95% confidence interval could be calculated based on the quantiles of the output distribution of Δ*g*_s_/Δ*c*_a_ (*η*_2*s*_), Δ*A*/Δ*c*_a_ (*γ*_2*s*_), and ΔiWUE/Δ*c*_a_ (*δ*_2*s*_). This statistical extension incorporates statistical uncertainty arising from the intraspecific variation in the *g*_smax_ into the model’s output.

#### 2.7.2. Empirical–Biochemical (EB) Simulation

The empirical–biochemical (EB) approach estimates iWUE values under light saturation and non-limiting vapour pressure deficit (VPD), which result in the maximum operating photosynthetic and *g*_s_ rates. This approach is also different from BiomeBGC* in the sense that it does not use average values for any parameter (i.e., species-specific parameterisation), but instead is based on individual values of the maximum rate of RuBisCO carboxylation (*V*_Cmax_) and *g*_s_ for each specimen (i.e., specimen-specific parameterisation). A dataset consisting of gas-exchange measurements, SLA, and N_area_ from 74 leaves belonging to 20 species (including the 5 species studied here) naturally growing in Fiji was used to develop an empirical model predicting the maximum rate of RuBisCO carboxylation at 25 °C (*V*_Cmax,25 °C_) (Supplementary data). These data were taken from a larger dataset generated by a set of measurements performed on 3–4 individuals per species in 2015, which was used in a separate publication [4]. Detailed descriptions of the gas-exchange measurement protocol have been provided elsewhere [4,23]. To develop the empirical model, firstly, the ‘one-point method’ was used to calculate the *V*_Cmax_ from the gas-exchange measurements, assuming that the day respiration (*R*_d_) was equal to 0.015 × *V*_Cmax_ [57]. Since the leaf temperature used in the gas-exchange measurements was higher than 25 °C, the temperature correction equations of [58] were used to calculate the *V*_Cmax,25_. Secondly, the statistical relationship between *V*_Cmax,25_ and the two functional traits, SLA and N_area_, were then established. The *V*_Cmax,25_, SLA, and N_area_ data were either ln-transformed (*V*_Cmax,25_ and SLA) or reciprocal-transformed (N_area_) to become normal. Fixed- and mixed-effects modelling approaches were used, and the model best describing *V*_Cmax,25 °C_ was determined based on the lowest Akaike information criterion value (AIC) and the normality of the model residuals. The selected mixed-effects model was of the following form:

(7) $$\ln V_{\mathrm{Cmax},25{}^{\circ}C}=a\cdot {\mathrm{recN}}_{\mathrm{area}}+b\cdot \mathrm{lnSLA}+c\cdot {\mathrm{recN}}_{\mathrm{area}}:\mathrm{lnSLA}+d+(1|\mathrm{Species})$$

The regression for Equation (7) had a total pseudo-R^2^ = 0.81. The model residuals were normally distributed (*p* = 0.066). The resultant empirical model was then used to calculate the *V*_Cmax,25_ of all of the herbarium leaf samples collected between 1927 and 2015, in conjunction with their SLA and N_area_ values. Therefore, the *V*_Cmax,25_ value was calculated only for the herbarium samples with a complete set of SLA and N_area_ values. Although our empirical model is based on data recorded on near-present-day plants, there is no clear evidence that the relationship linking *V*_Cmax_ with N_area_ and SLA changes over time, to our knowledge. For example, Walker et al. [59] demonstrated a global-scale relationship between *V*_Cmax_, N_area_, and SLA across various plant species. Although the potential change in the relationship between *V*_Cmax_, N_area_, and SLA through time is an interesting topic worthy of further investigation, we believe that, for the aim of our analysis, it is reasonable to assume that it does not.

Next, the operational light-saturated *g*_s_ values of the herbarium samples were estimated from their *g*_smax_ values. Previous studies have shown that, over developmental timescales and across plant lineages, changes in the operational *g*_s_ are mainly driven by corresponding changes in the density and size of stomata rather than dynamic stomatal opening adjustments, which are responsible for the short-term *g*_s_ feedback responses to environmental stimuli like the atmospheric CO_2_ concentration [60]. As a result, the ratio of *g*_s_ over *g*_smax_ should be relatively stable over developmental timescales, which is a common model assumption in deep-time reconstructions of *g*_s_ [61,62]. The *g*_s_/*g*_smax_ ratio was quantified by taking the ratio of the mean values of the light-saturated stomatal conductance to the water vapour (*g*_s_) measured in the field (see Ref. [23] for details) and the mean values of the *g*_smax_ inferred from the stomatal traits of 18 leaves belonging to all of the five species studied herein. The average *g*_s_/*g*_smax_ ratio was 0.51, and the *g*_s_ of the herbarium samples was calculated as *g*_s_ = 0.51*g*_smax_.

Following this, the photosynthetic rate (*A*) was calculated using the Farquhar–von Caemmerer–Berry photosynthesis model [63]. According to the model, the *A* at any intercellular CO_2_ concentration (*c*_i_) is given by the minimum of the RuBisCO-limited (*A*_c_) and RuBP regeneration-limited (*A*_j_) rates of CO_2_ assimilation, as follows:

(8) $$A=\min\left(A_{c},\;A_{j}\right)\;$$

(9) $$A_{c}=\frac{V_{\mathrm{Cmax}}\left(c_{i}-\Gamma^{*}\right)}{c_{i}+K_{C}(1+O/K_{O})}-R_{d}\;$$

(10) $$A_{j}=\frac{\left(c_{i}-\Gamma^{*}\right)J_{\max}/4}{c_{i}+2\Gamma^{*}}-R_{d}$$

where *K*_C_ and *K*_O_ are the Michaelis constants of RuBisCO for CO_2_ and O_2_, and Γ* is the CO_2_ compensation point in the absence or *R*_d_. *c*_i_ is needed to solve the system of Equations (8)–(10), and can be calculated from Fick’s law as follows:

(11) $$c_{i}=c_{a}-A/g_{t}$$

where *g*_t_ is the total conductance to the CO_2_ diffusion. Our *C*_i_ calculation does not take into account ternary effects due to the molecular interactions between H_2_O and CO_2_ molecules [64], and is used for the sake of simplicity. We consider it a reasonable simplification, since, upon inspection of the previously published gas-exchange data for the five species of the present study (see [4,23]), we found that not accounting for ternary interactions only resulted in a small 2% overestimation of the *C*_i_ values on average.

Assuming that the mesophyll conductance to CO_2_ diffusion (*g*_m_) is much higher than the stomatal conductance to CO_2_ diffusion (*g*_c_) and the boundary layer conductance to CO_2_ diffusion (*g*_cb_), the *g*_t_ of hypostomatous leaves can be calculated as follows:

(12) $$g_{t}=\frac{g_{c}\cdot g_{\mathrm{cb}}}{g_{c}+g_{\mathrm{cb}}}\;$$

For our calculations, a common *g*_cb_ of 2 mol m^−2^ s^−1^ was used for all of the samples, and the *g*_c_ was estimated as follows:

(13) $$g_{c}=\frac{g_{s}}{1.6}\;$$

where 1.6 is the ratio of the diffusivities of H_2_O and CO_2_ in the air.

Several lines of evidence suggest that, under saturating light and a *c*_a_ of 400 μmol mol^−1^ or lower, photosynthesis is limited by the activity of RuBisCO rather than the rate of ribulose 1,5-bisphosphate (RuBP) regeneration [65], as provided by Purcell et al., 2018. Accordingly, the substitution of *c*_i_ from Equation (11) in Equation (9) results in a quadratic equation, the positive root of which is the *A*. Our approach is very similar to that followed by Ethier and Livingston [66], with the only differences being the use of *c*_a_ and *g*_t_ in Equation (11) instead of ci and the internal conductance to CO2 (*g*_i_), respectively. For our calculations, the *V*_Cmax,25_ of the historical and modern samples and the temperature response of the published functions [58,67] were used to calculate the operational *V*_Cmax_, *K*_C_, *K*_O_, and Γ^*^ at 29 °C, which is the current mean leaf temperature (T_L_) in Fiji (see Ref. [23]). This assumes that recent increases in *c*_a_ have not resulted in significant increases in the operational T_L_. The iWUE of the herbarium samples was also calculated from the corresponding *A* and *g*_s_ values. Finally, linear regressions between each species samples’ *A*, *g*_s_, and iWUE vs. *c*_a_ at the time of sampling between 1927 and 2015 (Supplementary Data Figure S4, Supplementary Data Table S3) enabled the estimation of the Δ*g*_s_/Δ*c*_a_, Δ*A*/Δ*c*_a_, and Δ*iWUE*/Δ*c*_a_ coefficients for each species. The EB modelling is only applied to the herbarium samples with a complete set of the *g*_smax_, SLA, and N_area_ data.

#### 2.7.3. Comparison between the Results of Empirical Data, BiomeBGC Model (BiomeBGC*), and Empirical–Biochemical (EB) Simulations

The BiomeBGC* simulation requires an input of daily meteorological data, and the earliest availability for this data is from 1979 onwards. However, the empirical data and EB simulation cover the period between 1927 and 2015. Although the BiomeBGC* meteorological data did not cover the full range of the study period of the ~95 ppm *c*_a_ rise from 1927 to 2015, the analysis includes an important period of a steep *c*_a_ rise of ~65 ppm between 1979 and 2015 (Supplementary Data Figure S2a). This period is likely to bring the most impactful change in plant physiological response to the rising c_a_. Therefore, the comparison of the results amongst the three analyses is desirable because they cover the period of the steep *c*_a_ rise.

### 2.8. Statistical Analysis

All statistical analyses were undertaken using R statistical software [42]. Linear least-square regression was performed using the ‘lm’ function in R on all of the bivariate correlations. To determine whether the variance in the *g*_smax_ within each *c*_a_ range was larger than across *c*_a_, a one-way ANOVA was conducted on the *g*_smax_, which was binned into the following four *c*_a_ concentration ranges: 300–325 ppm, 326–350 ppm, 351–375 ppm, and 376–400 ppm.

## 3. Results

### 3.1. Leaf Stomatal Traits and iWUE Responses to Rising c_a_

**Figure 1:**
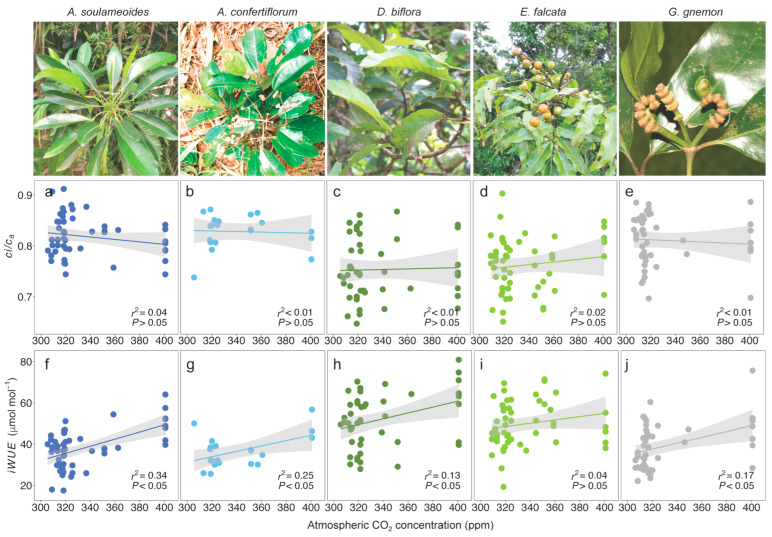
Ratio of leaf intercellular CO_2_ to ambient atmospheric CO_2_ (*c*_i_/*c*_a_. (**a**–**e**)) and intrinsic water-use efficiency (iWUE, (**f**–**j**)) responses to rising atmospheric CO_2_. Lines are the fitted regression. Shaded areas are the 95% confidence interval band.

**Figure 2:**
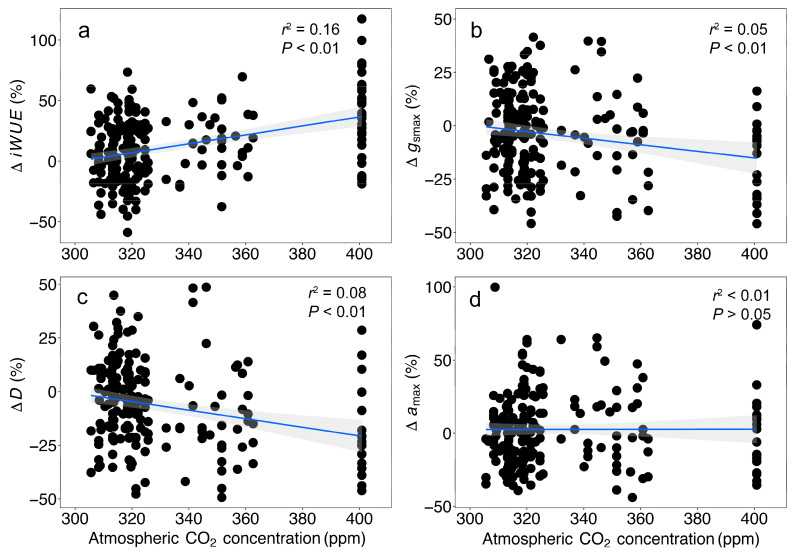
Relative sensitivity of traits to the atmospheric CO_2_ concentration (*c*_a_) of the combined species. (**a**) Intrinsic water-use efficiency (∆iWUE). (**b**) Maximum stomatal conductance (∆*g*_sma*x*_). (**c**) Stomatal density (∆*D*). (**d**) Maximum stomatal pore area (∆*a*_max_). Relative change is calculated as the percentage change to the intercept at 300 ppm c_a_. Lines are the fitted regression. Shaded areas are the 95% confidence interval band.

A lack of trend in the ratio of leaf intercellular CO_2_ (*c*_i_) to ambient atmospheric CO_2_ and *c*_i_/*c*_a_ with an increasing *c*_a_ was observed for all of the species (Figure 1a–e, Supplementary Data Table S4). There was a significant increase in the iWUE trends for four species between ~30% and 54%, while one species, *E. falcata*, did not show significant change (Figure 1f–j, Supplementary Data Table S4). The strongest iWUE response was in *A. soulameoides* at 0.17 µmol mol^−1^ ppm^−1^, while *D. biflora*, *G. gnemon*, and *A. confertiflorum* responded between 0.13 and 0.14 µmol mol^−1^ ppm^−1^. The overall iWUE response in a combined dataset was significantly positive at 0.14 µmol mol^−1^ ppm^−1^ and translated to an average iWUE increase of 37% per 100 ppm over the period of ~85 years (1930–1939 to 2015) (Figure 2a, Supplementary Data Table S5).

For each of the species, the range of δ^13^C values across *c*_a_ was considerably uniform when compared to the δ^13^C range at 400 ppm (i.e., the specimens collected by the authors in 2015) (Supplementary Figure S5). At 400 ppm, the δ^13^C ranges were as follows: *A. soulameoides*, 2.09 ‰ (–31.13 to –29.04‰); *A. confertiflorum*, 1.16‰ (–30.84 to –29.68‰); *D. biflora*, 3.53‰ (–31.13 to –27.60‰); *E. falcata*, 3.09‰ (–30.13 to –27.84‰); *G. gnemon*, 4.04‰ (–32.09 to –28.05‰).

**Figure 3:**
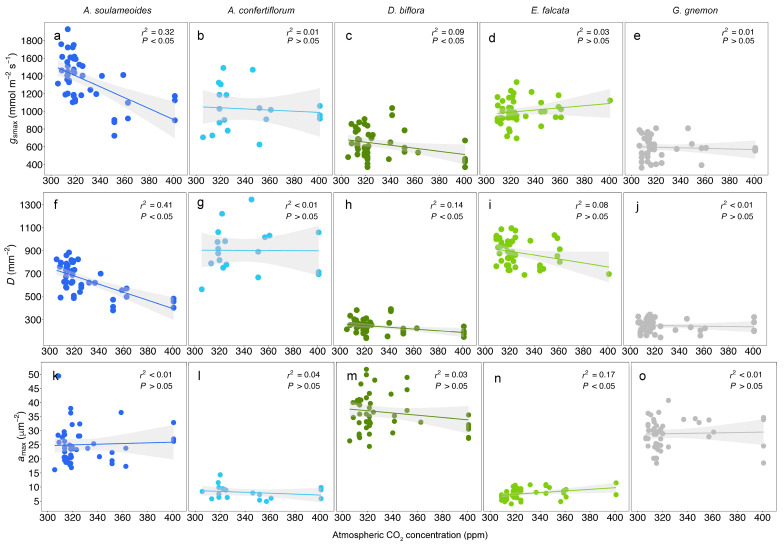
Species maximum stomatal conductance (*g*_smax_, (**a**–**e**)), stomatal density (*D*, (**f**–**j**)), and maximum stomatal pore area (*a*_max_, (**k**–**o**)) response to the rising atmospheric CO_2_ concentration. Lines are the fitted regression. Shaded areas are the 95% confidence interval band.

The species *g*_smax_ response was less consistent than the iWUE. With the rising *c*_a_, the *g*_smax_ decreased significantly in *A. soulameoides* and *D. biflora* at the rate of 6.18 mmol m^−2^ s^−1^ ppm^−1^ and 1.71 mmol m^−1^ s^−2^ ppm^−1^, respectively, but no significant trend was observed in the remaining three species (Figure 3a–e, Supplementary Data Table S6). *A. soulameoides* and *D. biflora* were also the only species that showed a significant reduction in stomatal density (*D*), at 3.52 mm^−2^ ppm^−1^ and 0.76 mm^−2^ ppm^−1^, respectively (Figure 3f–j, Supplementary Data Table S6). With respect to the maximum stomatal pore area (*a*_max_), only *E. falcata* showed a significant positive trend (Figure 3k–o)—an increase in *a*_max_ here may explain the lack of iWUE gain for *E. falcata* (Figure 1i). The general *g*_smax_ response in a combined dataset of the five species was significantly negative at −1.90 mmol m^−2^ s^−1^ ppm^−1^, and the overall *g*_smax_ relative sensitivity was significantly negative at 17% per 100 ppm (Figure 2b). This observed trend is mostly driven by *A. soulameoides* and *D. biflora.* The overall *D* relative sensitivity was significantly negative at −21% per 100 ppm (Figure 2c). The *a*_max_ relative sensitivity was weakly negative but not significant (Figure 2d). A comparison of the empirical iWUE and *g*_smax_ responses reveals several interesting trends. Two species, *A. soulameoides* and *D. biflora*, with a significant iWUE increase, also showed a significant *g*_smax_ decrease (Figure 1f,h and Figure 3a,c). On the contrary, *A. confertiflorum* and *G. gnemon*, with significant and relatively moderate iWUE increases, did not show a significant change in the *g*_smax_ (Figure 1g,j and Figure 3b,e). Lastly, *E. falcata* showed no significant trend in both the iWUE and *g*_smax_ (Figure 1i and Figure 3d). These findings suggest that *g*_smax_ is maybe influential in the iWUE responses (ΔiWUE/Δ*c*_a_), with a more negative *g*_smax_ response (Δ*g*_smax_/Δ*c*_a_) leading to a greater increase in the iWUE.

### 3.2. Phenotypic Space of a_max_ and D in Plant Communities

**Figure 4:**
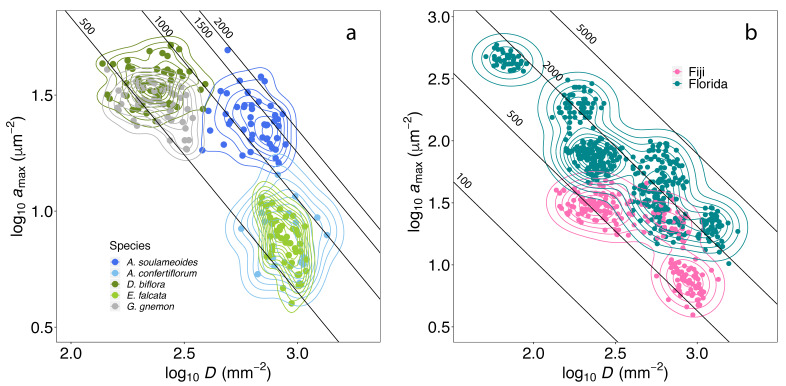
Contour plots showing the power law relationship between *a*_max_ and *D* in a logarithmic scale superimposed with the lines of equal *g*_sma*x*_; see Equation (1). (**a**) Clusters of five tree species in Fiji (*n* = 209). (**b**) Clusters of species from Florida (five species, *n* = 652) and Fiji coloured by location.

A contour plot of *a*_max_ and *D* with superimposed *g*_smax_ values (Figure 4) reveals how the *g*_smax_ values of individual species are constrained by the variability of the *a*_max_ and *D* within their phenotypic space. A total of three distinct clusters were formed by the species in this study. Two of the clusters were formed by a combination of *D. biflora–G. gnemon* and *A. confertiflorum–E. falcata*, which modulate within the same *g*_smax_ range of ~500–1000 mmol m^−2^ s^−1^ by having either a combination of high *a*_max_ and low *D* or low *a*_max_ and high *D*, respectively. *A. soulameoides* stands out as a separate cluster by adopting a higher *a*_max_ and *D* in modulating a higher *g*_smax_ range of ~1000–2000 mmol m^−2^ s^−1^ compared to the other species (Figure 4a). We superimposed our results with the published stomatal trait data from eight tree species in Florida [12,37], which shows that the phenotypic space of the aggregated species between the different biomes are also relatively dissimilar (Figure 4b), i.e., there is a low level of overlap between the trait distribution of the subtropical and tropical plant communities (a Jensen–Shannon divergence of 0.39; see Section 2). Interestingly, *A. soulameoides* is the only species that overlaps with one other species in Florida, *Myrica cerifera* L. Overall, our results indicate that species adopt different developmental strategies in modulating stomatal traits to alter the *g*_smax_, and the phenotypic space needed to achieve this may differ within and across plant communities.

### 3.3. The Effects of Extreme Climatic Conditions on iWUE

The iWUE data are not correlated with the self-calibrating Palmer Drought Severity Index (scPDSI), an index of drought (*r*^2^ = 0.01, *p* < 0.05, plot not shown). The iWUE is well-known to be affected by the *c*_a_ [30] and Mean Annual Precipitation (MAP) [68]. In this study, the multiple regression of the iWUE with *c*_a_ and MAP (*r*^2^ = 0.12, *p* < 0.05, plot not shown) shows that *c*_a_ contributes substantially to the variation in the iWUE, at 67%, compared to the MAP, at 33%, within the total contributed variation of 12%.

### 3.4. Variability in g_smax_

To assess the *g*_smax_ variability within and along *c*_a_, we performed an analysis of variance on the total *g*_smax_ data binned into four groups of a 25 ppm range (between 300 ppm and 400 ppm). The analysis shows that the within-group variation was higher than between-range-group variation (F (3, 203) = 2.03, *p* > 0.05)). This result suggests that much of the source variation in the *g*_smax_ data comes primarily from intraspecific variation rather than sampling gaps along *c*_a_.

### 3.5. Modelling Intrinsic Water-Use Efficiency Response (ΔiWUE/Δc_a_,), Stomatal Conductance Response (Δg_s_/Δc_a_), and Photosynthesis Response (ΔA/Δc_a_) to Rising c_a_, and Incorporating Intraspecific Variability of g_smax_

**Figure 5:**
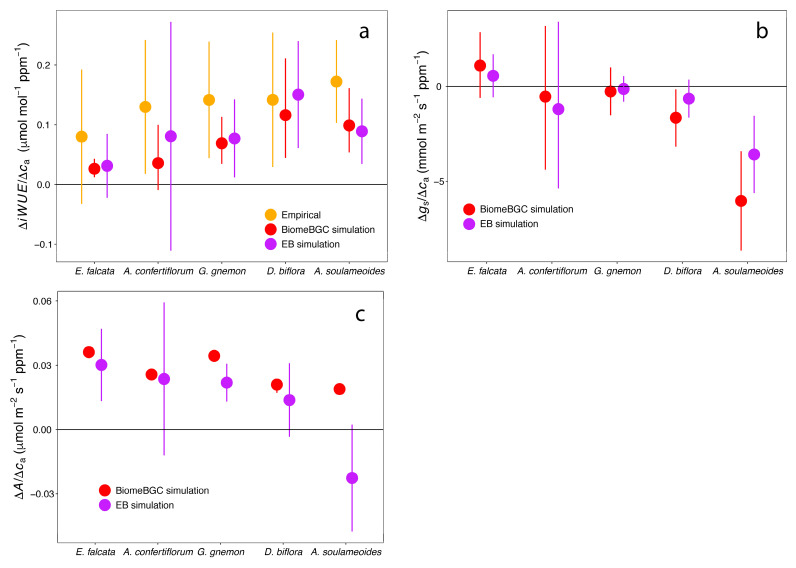
Simulation of species physiological trait responses to rising atmospheric CO_2_ concentration (*c*_a_) using the BiomeBGC (BiomeBGC^*^) and empirical–biochemical (EB) models. (**a**) Dotplot of the simulated intrinsic water-use efficiency responses (ΔiWUE/Δ*c*_a_) compared to the empirical results. (**b**) Dotplot of the simulated operational stomatal conductance response to the rising *c*_a_ (Δ*g*_s_/Δ/Δ*c*_a_). (**c**) Dotplot of the simulated photosynthesis responses to the rising *c*_a_ (Δ*A*/Δ*c*_a_). Dotplots representing the mean of simulated values and whiskers indicating the 95% confidence interval (CI), calculated based on the quantiles of the output distribution. See Supplementary Table S3 for details.

In examining the response of *g*_smax_ (Δ*g*_smax_/Δ*c*_a_) and its influence on the iWUE response (ΔiWUE/Δ*c*_a_) while accounting for intraspecific variations, we employed two models to simulate physiological responses. These models offer confidence intervals for the simulated traits. Our first model used a biogeochemical model (BiomeBGC) [52] with additional statistical steps, herein known as BiomeBGC* (Supplementary Data Figure S3). This approach incorporated statistical uncertainty arising from the intraspecific variation in the *g*_smax_, which varies annually. The BiomeBGC* model used species-specific parameterisation and an estimates average, the aggregated values of the simulated iWUE, *A* and *g*_s_ for all times of day, and all changes in environmental variables. The second model was an empirical–biochemical (EB) model that simulated the iWUE, *A*, and *g*_s_ from each individual empirical data point under light-saturation and non-limiting VPD. The EB modelling approach was also different from BiomeBGC*, in that it used specimen-specific instead of species-specific parameterisation. In both of these models, the mean simulated ΔiWUE/Δ*c*_a_ values at the species level were in general agreement with the empirical trend (RMSEs of 0.019 and 0.023), but were, on the average, half of the magnitude of the empirical responses (Figure 5a). The two models were in agreement with the empirical data, in that *A. soulameoides* and *D. biflora* were ranked as the top two species, with the highest simulated ΔiWUE/Δ*c*_a_, while the lowest ranked species was *E. falcata* (Figure 5a). These top two species were also the only species showing significant decreases in the BiomeBGC* simulated Δ*g*_s_/Δ*c*_a_ (Figure 5b), while the other three species (*Astronidium confertiflorum, Elattostachys falcata*, and *Gnetum gnemon*) displayed no significant trends. All of the species showed significant increases in the BiomeBGC* simulated photosynthesis response (Figure 5c). However, the Δ*g*_s_/Δ*c*_a_ and Δ*A*/Δ*c*_a_ from the EB model varied, with a mostly insignificant change, and these were an artefact of the incomplete data in the time series. For example, the 95% confidence intervals for the traits simulated in *A. confertiflorum* are very wide (Figure 5a–c, Supplementary Data Table S7) because of the absence of a data point at 400 ppm *c*_a_ (Supplementary Data Figure S4). The application of the EB model is limited to herbarium samples with a complete set of *g*_smax_, specific leaf area (SLA), and leaf nitrogen per area (N_area_) data. Taken together, our BiomeBGC* simulations and empirical results suggest a more pronounced influence of *g*_smax_ responses (Δ*g*_smax_/Δ*c*_a_) in determining the direction of iWUE responses (ΔiWUE/Δ*c*_a_) when Δ*A*/Δ*c*_a_ shows a general consistent increase across species, i.e., a weaker *g*_smax_ response (Δ*g*_smax_/Δ*c*_a_) leading to a greater increase in the iWUE.

## 4. Discussion

For the first objective of this study, we showed a trend of iWUE gain in four out of five species associated with a contemporaneous increase in *c*_i_ with *c*_a_ (Figure 1a–e). The conservatism in the ratio of *c*_i_/*c*_a_ has been reported in many studies [69], but not in all species [4]. The proportional regulation of carbon gain and water loss by the modulation of *A* and *g*_s_, respectively, maximises the overall carbon gain and minimises the water loss, which resulted in an improved iWUE [1]. For the species with a significant iWUE gain, i.e., between ~30% and 54% (Figure 1f–j), our result is in agreement with previous studies which show an iWUE gain between ~25% and 52% in seven tree species for a period of approximately a century in the tropics [3,70,71]. The overall iWUE increase of 37% over the period of ~85 years (1930–1939 to 2015) (Figure 2a) reinforced the findings in other studies, which showed iWUE gains of 30–35% in the tropics [72] and ~40% worldwide [73] spanning over a century. Non-responsive iWUE trends to rising *c*_a_, although not predominant, have been recorded in many studies, e.g., a recent review found that 16% of trees (18 of 113 trees) investigated did not show any trends for the recent half of the century [73]. Previous research has indicated that a non-responsive iWUE trend in temperate trees is associated with an increase in guard cell length, a trait tightly linked to *a*_max_ [74]. Our result for *E. falcata* supported this finding and is the only known documented observation in a tropical tree. A further species-specific physiological study is needed to understand the reason behind this observation.

For each species, we found a wide and consistent range of δ^13^C values in both historical herbarium specimens and recent collections from 2015 (i.e., samples collected by the authors using a standardised protocol). This indicates that factors such as elevation, canopy height, leaf morphotype, and herbarium preservation techniques may not significantly affect the δ^13^C variability in our samples. In comparison, a different study of the leaf δ^13^C from 64 tree species in the French Guiana tropical rainforest documented a wider range of δ13C values for sunlit leaves, with a species-averaged range of approximately 7.3‰ (–34.8 to –27.5‰) [75].

In contrast to a study involving nine subtropical tree species in Florida [12], our study demonstrates an overall *g*_smax_ response of −17% per 100 ppm, which is nearly half of the response observed in the Florida study (−33% per 100 ppm). However, the more striking result to emerge from this study and from Lammertsma et al. [12] is that the *D* relative sensitivity is greater than *a*_max_, which suggests that *D* is more influential than *a*_max_ in determining the *g*_smax_ response to *c*_a_ in these tropical and subtropical taxa. There are only a few long-term studies on stomatal micromorphological trait responses to changing *c*_a_ in the tropics. For example, a study on two Amazonian tree species did not find any changes in the stomatal surface area (a trait correlated with *g*_smax_) and *D* for over a century of *c*_a_ rise from 280 ppm to 380 ppm [3]. Taken together, there is evidence to suggest that the direction of *D* responses to rising *c*_a_ in the tropics are variable and species-specific. This is contrary to the temperate ecosystem, where *D* has been observed to decrease significantly in most C3 woody plants between the early 21st century and the recent decades, with a *c*_a_ rise of ~100 ppm [11].

The large variation in the stomatal traits (*a*_max_ and *D*) and, subsequently, *g*_smax_ values, reflect the high intraspecific trait variability in these traits (Figure 4). It was observed here, and in many studies [76], that *g*_smax_ is constrained by the variability of *a*_max_ and *D*, with individual species forming clusters along the *a*_max_ and *D* power law relationship. These clusters may partly represent species-specific phenotypic plasticity [12,14], herein developmental plasticity, which relates to the capacity to change *g*_smax_ through both developmental change in *D* and *a*_max_. The evidence presented in this paper suggests the species-specific implementation of developmental strategies in modulating *a*_max_ and *D* to modify *g*_smax_. Consequently, this had necessitated coexisting species to occupy a specific phenotypic space within the Fijian plant communities, forming specific clusters (Figure 4a). This observation lends support to the concept of ecophysiological niche segregation [15,77] within plant communities. The minimal overlap in the probability distribution of stomatal traits between the plant communities in Fiji and Florida (Figure 4b) implies different developmental strategies adopted in modulating stomatal traits to alter the *g*_smax_ by the two plant communities. Further study is required to better understand the reason behind the ecophysiological niche space amongst coexisting plant species in the complex tropical ecosystem, and how this may confer higher competitive ability and climate resilience.

The high data variability, which stems from a combination of intraspecific trait variations and sampling gaps in herbarium samples, has consequently led to weak correlations in most of the bivariate relationships in this study, with low-to-moderately high *r*^2^ values ranging between ~0.01 and 0.32. Our analysis shows that much of the variance in the *g*_smax_ data is attributed to intraspecific variation rather than sampling gaps in the herbarium specimens. For example, in this study, the *D* and *a*_max_ data, and, accordingly, the *g*_smax_, vary between ±25% and ±50% from the fitted regression value for the same c_a_. (Figure 3). Similar high stomatal trait variability was also observed in previous studies in subtropical and tropical trees [3,12]. As a result, the large confidence intervals in the empirical iWUE response of each species (ΔiWUE/Δ*c*_a_) (Figure 5a) is also the consequence of high data variability.

The second objective of this study sought to investigate the influence of *g*_smax_ and its intraspecific variation on the iWUE over rising c_a_. Our results showed that the trend of the simulated ΔiWUE/Δ*c*_a_ generally agreed with empirical data and differed from the latter in lower magnitude. The propensity for vegetation models to underestimate water-use efficiency gains is well documented because of the various assumptions made about the processes influencing the iWUE [78]. Recent evidence showed widespread global increases in *A* as a result of rising *c*_a_ driving the iWUE gain [73,79,80]. While our simulation results indicate that *A* is the main factor contributing to the iWUE gain (Figure 5), *g*_s_ is driving the trend in the iWUE gain across tree species. Our modelling approach is important for assessing empirical results and providing an opportunity to explore covariation, because the variations in empirical data can mask the species *g*_smax_ and iWUE responses to rising c_a_. To the best of our knowledge, there are no other vegetation models that incorporate the intraspecific variation in the *g*_smax_. Our method enables confidence intervals derived from the intraspecific variation in *g*_smax_ to be placed on the modelled iWUE and *g*_s_, and therefore providing an informed assessment on the significance of the average value. This standalone statistical extension can be readily implemented in other vegetation models without modification to the original model. Our approach loosely falls within the framework of the ‘plastic model of intraspecific variation’ category type of trait-flexible models [81,82], which requires an input of the relationship between a trait and its environment rather than a single mean trait value. Additionally, the use of species-specific and specimen-specific parameterisations in the BiomeBGC* and EB models, respectively, has also improved our prediction, and we therefore recommend integrating specific information in the modelling to improve the simulation of physiological traits. This highlights the importance of gathering specimen- or species-specific physiological and functional trait data for model parameterisation.

The iWUE response observed in this study may have been intensified by the frequent El Niño-induced drought from the 1970s to 2015. During this period, three record-strong El Niño events were documented in 1982–1983, 1997–1998, and 2015–2016 [51]. Drought may lead to a greater iWUE response, as certain species with responsive stomatal closing and opening rates in response to instantaneous leaf water availability may increase the carbon fixed per unit water loss, thereby improving the chance of survival in drier environments [83]. The effects of elevated *c*_a_ on plants under drought or in arid conditions are complex and depend on the severity of the drought stress [83]. Hence, quantifying the contribution of *c*_a_ and drought stress to elevated iWUE is not straightforward—for example, our iWUE data are not correlated with drought. The iWUE is well known to be affected by the *c*_a_ [30] and MAP [68]. In this study, we showed that *c*_a_ largely explains the variation in the iWUE more than the MAP. This is further supported by the results of the simulated iWUE in both the BiomeBGC* and EB models, which are notably similar, notwithstanding that the earlier model interacts with daily meteorological variables in Fiji but the latter is does not.

The small island nations of the Pacific region, although contributing only a fraction of global greenhouse gas emissions, will encounter disproportionate consequences from global climate change [84]. The region harbours many of the world’s biodiversity hotspots, e.g., the four out of five species studied here are endemic to the Southwest Pacific islands. Many of the endemic plant species are susceptible to and already impacted by many effects of anthropogenic climate change, such as sea level rise, ocean acidification, changes in rainfall and temperature, and more extreme weather events [84]. Past studies of the climate change impact on vegetation in the Pacific region have been largely focused on species range shift [84]. However, to date, little is known about the long-term physiological response of the native plants to climate change in this region, particularly their response to rising c_a_. This knowledge is important, because land plants are a critical component in biosphere–atmosphere processes [85,86]. For example, plants, through photosynthesis and transpiration, couple the carbon and water cycles, thereby playing a pivotal role in the Earth system and plant–climate feedbacks [5,76,87,88]. In this regard, the impact of plant physiological response to rising *c*_a_ at ecosystem scale can have a profound effect on island nations in the Pacific region, considering the high forest cover in the region, e.g., 52.6% of Fiji’s landmass is covered by forest [89].

## 5. Conclusions

Our results highlight the species-specific physiological responses to the decadal period of climate change among five tropical tree species in the Pacific region. While certain species demonstrate discernible trends, others show no apparent patterns. However, the overall trend supported by empirical and simulated results points to the more pronounced influence of *g*_smax_ on the direction of the iWUE response to rising *c*_a_ under a trend of gain in *A*—a general trend of a stronger increase in the iWUE response with a stronger decline in *g*_smax_, and vice versa. This plastic response in the *g*_smax_ of individual species to changing *c*_a_, as it relates to the iWUE as an outcome, is also a characteristic of species resilience and competitiveness. As the variation in a trait increases, the detection of responses to environmental variables becomes more difficult due to the increased probability of committing Type II statistical errors. Modelling helps to incorporate variability in plant traits, such as the *g*_smax_ related to the response of physiological attributes to changes in environmental conditions, and highlights the need for specification rather than generalisation when applied to larger regions. This study also provides an insight on the little-known CO_2_-induced tree physiological response in the Pacific Islands, a region that is most susceptible to the impact of global warming.

## Data Availability

Data and supplementary images and tables are available at *Biology* online. Codes for the simulation are available at https://github.com/jelkink/fiji. (accessed on 18 September 2024).

## Supplementary Materials

The following supporting information can be downloaded at: https://www.mdpi.com/article/10.3390/biology13090733/s1, Figure S1: Leaf cuticle impression taken at 400× magnification showing the stomata of the species studied; Figure S2: Climate trends from 1927 to 2015 in Fiji; Figure S3: Flowchart statistical procedures in the BiomeBGC simulations of ΔiWUE/Δ*c*_a_, Δ*g*_s_/Δ/Δ*c*_a_, and Δ*A*/Δ*c*_a_; Figure S4: Intrinsic water-use efficiency, stomatal conductance, and photosynthesis simulated from the empirical–biochemical (EB) model of the sampled herbarium leaves plotted against the atmospheric CO_2_ concentration; Figure S5: The δ^13^C of the sampled herbarium leaves plotted against the atmospheric CO_2_ concentration; Table S1: Parameterisation for the BiomeBGC* simulation; Table S2: Species-specific parameters for the BiomeBGC* simulation; Table S3: Empirical–biochemical (EB) simulation results, and the linear regression of *g*_s_ (mmol m^−2^ s^−1^), *A* (µmol m^−2^ s^−1^), and iWUE (µmol mol^−1^) versus *c*_a_ (ppm); Table S4: Linear regression of δ^13^C (per mille), *c*_i_/*c*_a_, and iWUE (µmol mol^−1^) versus *c*_a_ (ppm); Table S5: Relative sensitivities (% ppm^−1^) in iWUE, *g*_smax_, *D*, and *a*_max_ of the total dataset to the *c*_a_ (ppm) increase; Table S6: Linear regression of *g*_smax_ (mmol m^−1^ s^−2^), *D* (mm^−2^), and *a*_max_ (µm^−2^) versus *c*_a_ (ppm); Table S7: Mean values and 95% confidence interval (CI_95%_) of empirical and simulated intrinsic water-use efficiency response (ΔiWUE/Δ*c*_a_), operational stomatal conductance response (Δ*g*_s_/Δ/Δ*c*_a_), and photosynthesis response (Δ*A*/Δ*c*_a_).
