# Supplementary material for: Stomata Are Driving the Direction of CO2-Induced Water-Use Efficiency Gain in Selected Tropical Trees in Fiji
Source: Biology (Basel). 2024 Sep 19;13(9):733. doi: 10.3390/biology13090733 (PMC11428275; doi:10.3390/biology13090733)
Supplement: Supplementary file 1 [file biology-13-00733-s001.zip › Supplementary figures and tables MDPI.pdf]

Supplementary Information: figures and tables

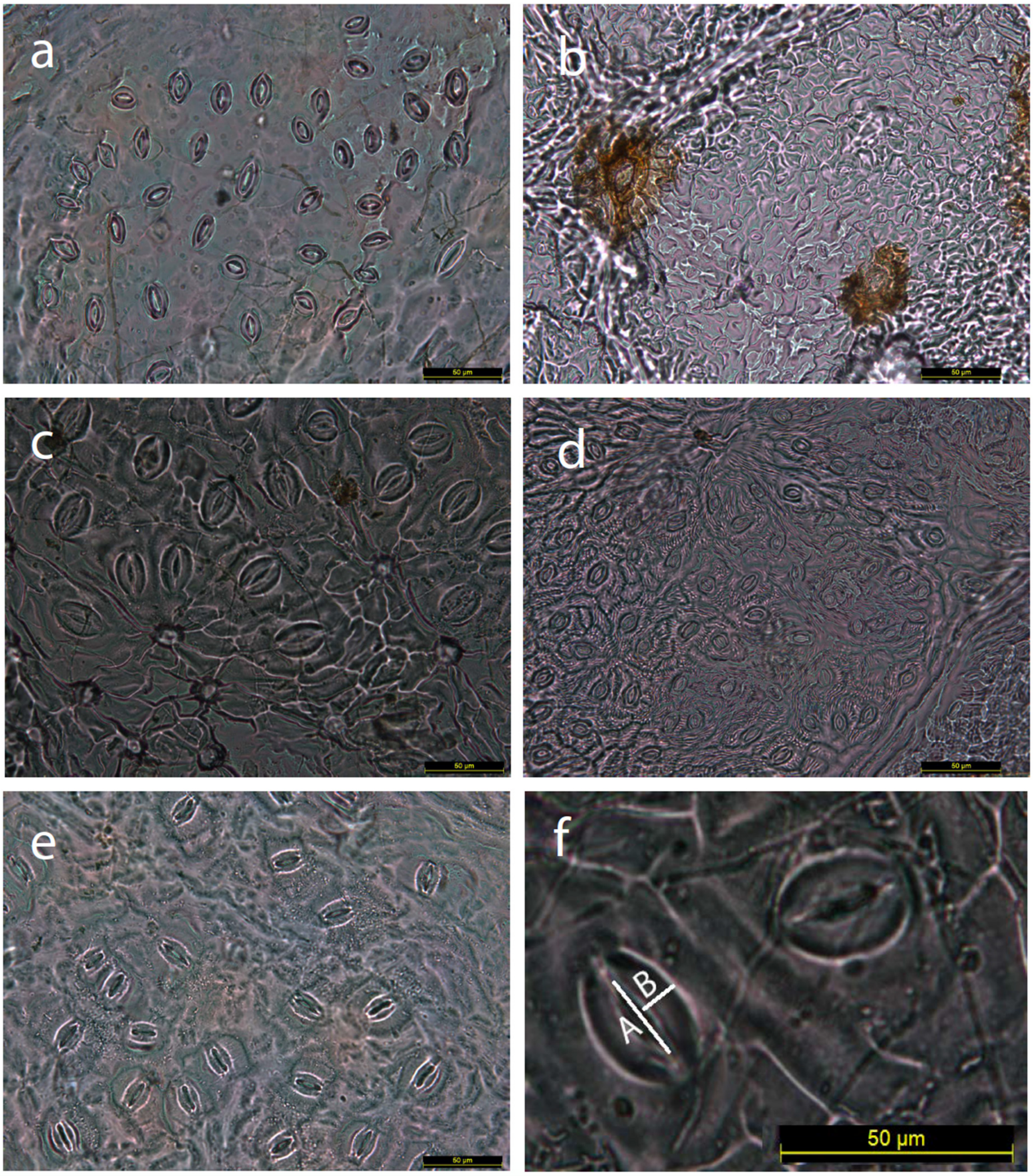

Fig. S1. Leaf cuticle impression taken at 400x magnification showing stomata of the species studied.(a) *Amaroria soulameoides*. (b) *Astronidium confertiflorum.* (c) *Dillenia biflora.* (d) *Elattostachys falcata*. (e) *Gnetum gnemon.* (f) Stomata of *Dillenia biflora* showing the measured pore length (A) and guard cell width (B). Scale bar is 50 µm.

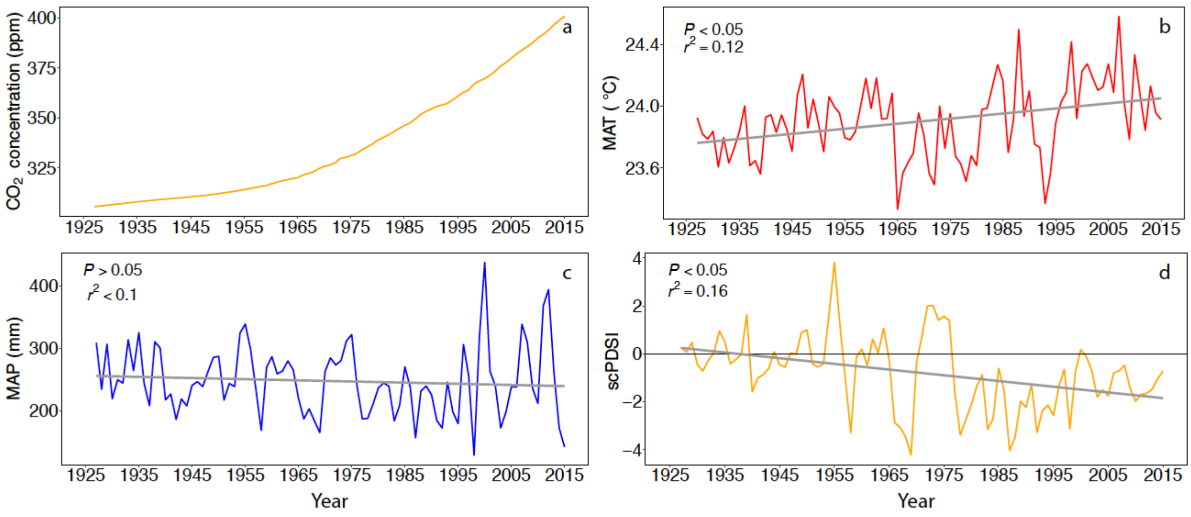

Fig. S2. Climate trends from 1927 to 2015 in Fiji. (a) Atmospheric CO_2 concentration, c_a (ppm). (b) Mean annual temperature (MAT). (c) Mean annual precipitation (MAP). (d) Drought index, self-calibrating Palmer Drought Severity Index (scPDSI). Grey lines are the fitted regression.

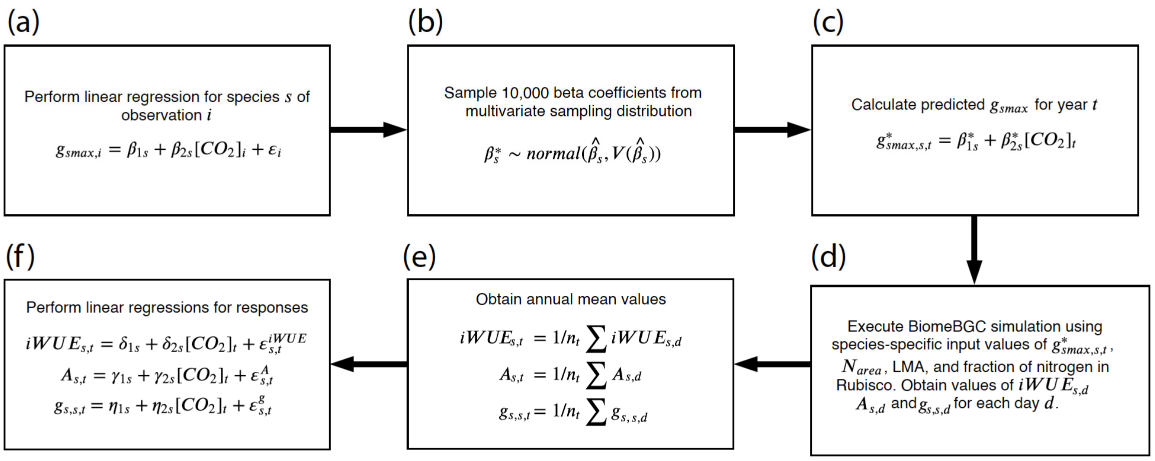

Fig. S3. Flowchart statistical procedures in BiomeBGC simulations of ΔiWUE/Δc_a, Δg_s/Δ/Δc_a and ΔA/Δc_a. (a–f). Variables β, δ, γ and η are the model coefficients and ε is the model residual for species s. n_t represents the total number of days for year t. The values of δ_{2s}, γ_{2s} and η_{2s} together with their confidence intervals are shown in Fig. 5.

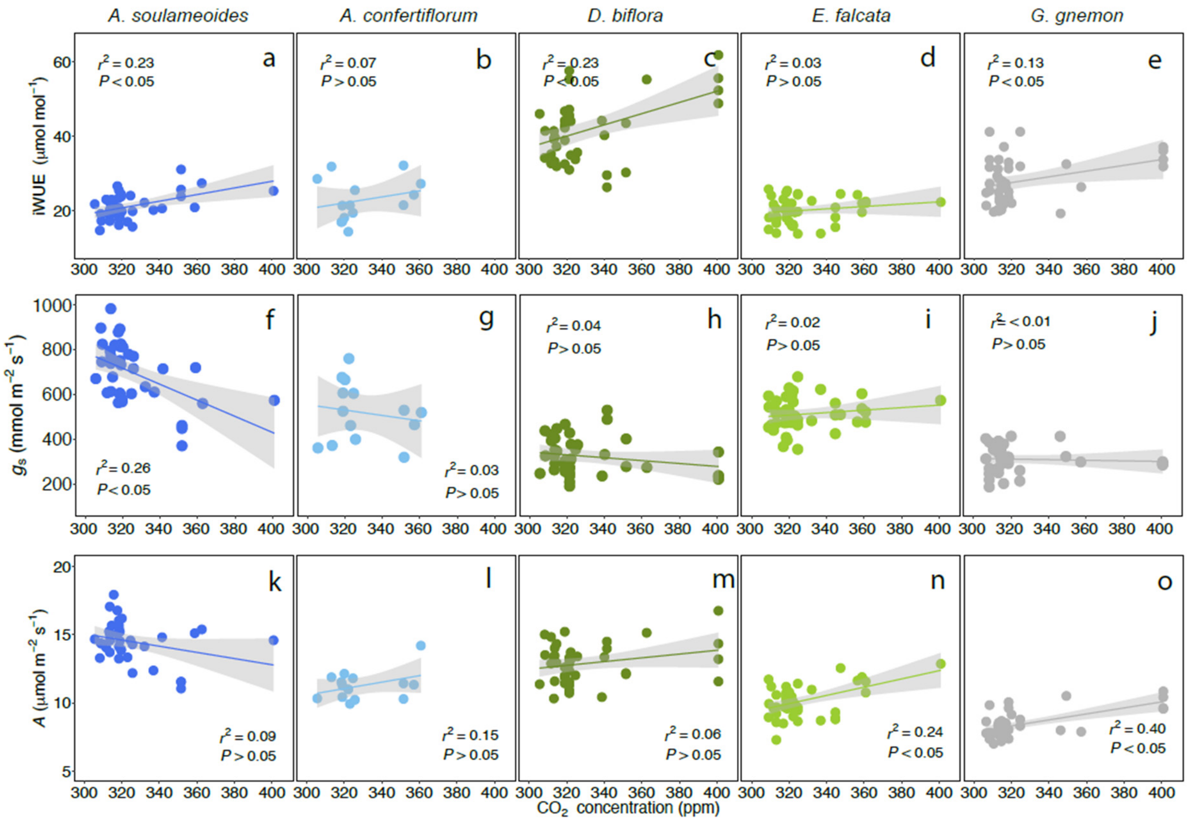

Fig. S4. Intrinsic water-use efficiency (iWUE, a–e), stomatal conductance (g_s, f–j) and photosynthesis (A, k–o) simulated from Empirical-biochemical (EB) model of sampled herbarium leaves plotted against atmospheric CO_2 concentration. Lines are the fitted regression. Shaded areas are the 95% confidence interval band.

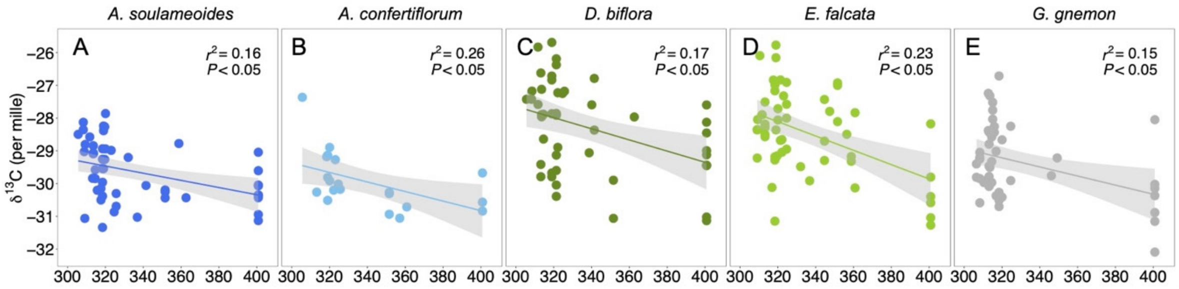

Fig. S5. $\delta^{13}C$ of sampled herbarium leaves plotted against atmospheric CO_2 concentration. Lines are the fitted regression. Shaded areas are the 95% confidence interval band.

Table S1. Parameterisation for BiomeBGC* simulation.

Parameters/input	Data source	Period	Note
Daily meteorological data	CPC Global Daily Gridded (0.5° x 0.5°)	1979-2015	BiomeBGC requires an input of daily meteorological data. The earliest availability for this data is 1979.
LMA average (g m^{-2})	Herbarium specimens (n =216)	1927-2015	A typical BiomeBGC simulation requires an input of a single value for each these parameter. A single average value of each trait, for the period of 1927-2015, were used as parameter. Because there are no significant
SLA (m^2 kg C)	Herbarium specimens (n = 216)	1927-2015	

N_{area} average (g m-2)	Herbarium specimens (n = 216)	1927-2015	trends in these traits across time (1927-2015, P > 0.05), it is therefore reasonable to estimate these parameters using averages of the whole dataset.
C:N	Herbarium specimens (n = 216)	1927-2015	
BGC VCmax,25°C (umol m-2 s-1)	Calculated using average LMA and C:N above, following Thornton and Running, 2002		
Fraction of leaf nitrogen in RuBisCO	Estimated from BiomeBGC simulation as such that simulated *A* is close to average empirical *A*. The average empirical *A* values for each species were obtained from IRGA measurements of living specimens in their natural habitat in Fiji from a study conduced in 2015 (see Supplementary data) (Soh et al. 2019).		

Table S2. Species-specific parameters for BiomeBGC* simulation.

Species	LMA average (g m^{-2})	SLA (m^2 kg C)	N_{area} average (g m^{-2})	C:N leaf	BGC $V_{Cmax,25°C}$ (umol m^{-2} s^{-1})	Fraction of leaf nitrogen in RuBisCO
Amaroria soulameoides	89.85	22.3	1.93	23.3	38.7	0.145
Astronidium confertiflorum	110.99	18.0	1.74	31.9	34.9	0.080
Dillenia biflora	92.62	21.6	1.64	28.2	32.9	0.130
Elattostachys falcata	92.36	21.7	2.13	21.7	42.7	0.035
Gnetum gnemon	99.57	20.1	2.54	19.6	50.9	0.030

Table S3. Empirical-biochemical (EB) simulation results, Linear regression of g_s (mmol m^{-2} s^{-1}), A (µmol m^{-2} s^{-1}) and iWUE (µmol mol^{-1}) versus c_a (ppm).

Trait	Species	Sample size	Function	r^2	*P*-value
iWUE	*Amaroria soulameoides*	38	iWUE = $0.09 \cdot c_a - 7.63$	0.23	0.05 <
	Astronidium confertiflorum	14	iWUE = $0.08 \cdot c_a - 3.68$	0.07	> 0.05
	Dillenia biflora	41	iWUE = $0.15 \cdot c_a - 8.01$	0.23	0.05 <
	Elattostachys falcata	44	iWUE = $0.03 \cdot c_a + 9.97$	0.03	> 0.05
	Gnetum gnemon	41	iWUE = $0.08 \cdot c_a + 2.99$	0.13	0.05 <
g_s	*Amaroria soulameoides*	38	$g_s = -3.59 \cdot c_a + 1864$	0.26	0.05 <
	Astronidium confertiflorum	14	$g_s = -1.2 \cdot c_a + 915$	0.03	> 0.05
	Dillenia biflora	41	$g_s = -0.65 \cdot c_a + 537$	0.04	> 0.05
	Elattostachys falcata	44	$g_s = 0.56 \cdot c_a + 329$	0.02	> 0.05
	Gnetum gnemon	41	$g_s = -0.13 \cdot c_a + 354$	4.21E-03	> 0.05
A	*Amaroria soulameoides*	38	$A = -0.02 \cdot c_a + 21.83$	0.09	> 0.05
	Astronidium confertiflorum	14	$A = 0.02 \cdot c_a + 3.47$	0.15	> 0.05
	Dillenia biflora	41	$A = 0.01\ c_a + 8.33$	0.06	> 0.05
	Elattostachys falcata	44	$A = 0.03 \cdot c_a + 0.27$	0.24	0.05 <
	Gnetum gnemon	41	$A = 0.02 \cdot c_a + 1.29$	0.4	0.05 <

Table S4. Linear regression of $\delta^{13}C$ (per mille), c_i/c_a and iWUE (µmol mol^{-1}) versus c_a (ppm).

Trait	Species	Sample size	Function	r^2	*P*-value
$\delta^{13}C$	*Amaroria soulameoides*	51	$\delta^{13}C = -0.01 \cdot c_a + -25.97$	0.16	3.8E-3
	Astronidium confertiflorum	20	$\delta^{13}C = -0.01 \cdot c_a + -25$	0.26	0.02
	Dillenia biflora	47	$\delta^{13}C = -0.02 \cdot c_a + -22.53$	0.17	4.6E-3
	Elattostachys falcata	53	$\delta^{13}C = -0.02 \cdot c_a + -21.12$	0.23	3.1E-4
	Gnetum gnemon	45	$\delta^{13}C = -0.01 \cdot c_a + -24.77$	0.15	0.01

c_i/c_a	*Amaroria soulameoides*	51	$c_i/c_a = 2.4E\text{-}4 \cdot c_a + 0.9$	0.34	7.7E-7
	Astronidium confertiflorum	20	$c_i/c_a = -5.6E\text{-}5 \cdot c_a + 0.85$	0.25	0.03
	Dillenia biflora	47	$c_i/c_a = 5.9E\text{-}5 \cdot c_a + 0.73$	0.13	0.01
	Elattostachys falcata	53	$c_i/c_a = 2.6E\text{-}4 \cdot c_a + 0.67$	0.04	0.16
	Gnetum gnemon	45	$c_i/c_a = -1.1E\text{-}4 \cdot c_a + 0.85$	0.17	0.01
iWUE	*Amaroria soulameoides*	51	iWUE = $0.172 \cdot c_a + 19.6$	0.34	7.7E-7
	Astronidium confertiflorum	20	iWUE = $0.130 \cdot c_a + -7.6$	0.25	0.03
	Dillenia biflora	47	iWUE = $0.142 \cdot c_a + 4.1$	0.13	0.01
	Elattostachys falcata	53	iWUE = $0.080 \cdot c_a + 22.9$	0.04	0.16
	Gnetum gnemon	45	iWUE = $0.141 \cdot c_a + -7.7$	0.17	0.01

Table S5. Relative sensitivities (% ppm^{-1}) in iWUE, g_{smax}, D, and a_{max} of total dataset to c_a (ppm) increase.

Trait	Sample size	Relative sensitivity (% ppm^{-1})	r^2	P-value
iWUE	216	0.37	0.16	7.8E-10
g_{smax}	209	0.17	0.05	1.6E-3
D	209	-0.21	0.08	6.3E-5
a_{max}	209	-0.0018	3.6E-6	0.98

Table S6. Linear regression of g_{smax} (mmol m^{-1} s^{-2}), D (mm^{-2}) and a_{max} (μm^{-2}) versus c_a (ppm).

Trait	Species	Sample size	Function	r^2	P-value
g_{smax}	*Amaroria soulameoides*	46	$g_{smax} = -6.18 \cdot c_a + 3380$	0.32	3.6E-5
	Astronidium confertiflorum	19	$g_{smax} = -0.65 \cdot c_a + 1248$	0.01	0.74
	Dillenia biflora	50	$g_{smax} = -1.71 \cdot c_a + 1199$	0.09	0.03
	Elattostachys falcata	47	$g_{smax} = 1.09 \cdot c_a + 641$	0.03	0.23
	Gnetum gnemon	47	$g_{smax} = -0.32 \cdot c_a + 698$	0.01	0.62
D	*Amaroria soulameoides*	46	$D =- -3.52 \cdot c_a + 1806$	0.41	1.6E-6
	Astronidium confertiflorum	19	$D = -0.05 \cdot c_a + 921$	0.7E-5	0.97
	Dillenia biflora	50	$D = -0.76 \cdot c_a + 493$	0.14	0.01
	Elattostachys falcata	47	$D = -1.71 \cdot c_a + 1443$	0.08	0.06
	Gnetum gnemon	47	$D = -0.13 \cdot c_a + 290$	3.9E-3	0.68
a_{max}	*Amaroria soulameoides*	46	$a_{max}=0.01 \cdot c_a + 21$	2.0E-3	0.76
	Astronidium confertiflorum	19	$a_{max}=-0.02 \cdot c_a + 14$	0.04	0.39
	Dillenia biflora	50	$a_{max}=-0.04 \cdot c_a + 50$	0.03	0.25
	Elattostachys falcata	47	$a_{max}=0.03 \cdot c_a + -2$	0.12	0.02
	Gnetum gnemon	47	$a_{max}=0.01 \cdot c_a + 27$	1.4E-3	0.80

Table S7. Mean values and 95% confidence interval ($CI_{95\%}$) of empirical and simulated intrinsic water-use efficiency response (ΔiWUE/Δc_a), operational stomatal conductance response ($\Delta g_s/\Delta/\Delta c_a$) and photosynthesis response ($\Delta A/\Delta c_a$).

Species	Trait response	Mean	$CI_{95\%}$	Sample size	Model/data source
Amaroria soulameoides	ΔiWUE/Δc_a	0.172	[0.103, 0.242]	51	Empirical
Astronidium confertiflorum	ΔiWUE/Δc_a	0.130	[0.018, 0.242]	20	Empirical

Dillenia biflora	ΔiWUE/Δc_a	0.142	[0.029, 0.254]	47	Empirical
Elattostachys falcata	ΔiWUE/Δc_a	0.080	[-0.032, 0.193]	53	Empirical
Gnetum gnemon	ΔiWUE/Δc_a	0.141	[0.044, 0.239]	45	Empirical
Amaroria soulameoides	ΔiWUE/Δc_a	0.099	[0.054, 0.161]	10,000	BiomeBGC*
Astronidium confertiflorum	ΔiWUE/Δc_a	0.036	[-0.009, 0.1]	10,000	BiomeBGC*
Dillenia biflora	ΔiWUE/Δc_a	0.116	[0.045, 0.211]	10,000	BiomeBGC*
Elattostachys falcata	ΔiWUE/Δc_a	0.026	[0.012, 0.043]	10,000	BiomeBGC*
Gnetum gnemon	ΔiWUE/Δc_a	0.069	[0.035, 0.113]	10,000	BiomeBGC*
Amaroria soulameoides	ΔiWUE/Δc_a	0.089	[0.035, 0.144]	38	EB
Astronidium confertiflorum	ΔiWUE/Δc_a	0.081	[-0.111, 0.272]	14	EB
Dillenia biflora	ΔiWUE/Δc_a	0.150	[0.061, 0.24]	41	EB
Elattostachys falcata	ΔiWUE/Δc_a	0.031	[-0.022, 0.085]	44	EB
Gnetum gnemon	ΔiWUE/Δc_a	0.077	[0.012, 0.142]	41	EB
Amaroria soulameoides	$\Delta g_s/\Delta c_a$	-6.032	[-8.647, 3.415]	10,000	BiomeBGC*
Astronidium confertiflorum	$\Delta g_s/\Delta c_a$	-0.539	[-4.386, 3.18]	10,000	BiomeBGC*
Dillenia biflora	$\Delta g_s/\Delta c_a$	-1.654	[-3.174, 0.158]	10,000	BiomeBGC*
Elattostachys falcata	$\Delta g_s/\Delta c_a$	1.095	[-0.606, 2.846]	10,000	BiomeBGC*
Gnetum gnemon	$\Delta g_s/\Delta c_a$	-0.270	[-1.524, 0.998]	10,000	BiomeBGC*
Amaroria soulameoides	$\Delta g_s/\Delta c_a$	-3.585	[-5.621, 1.55]	38	EB
Astronidium confertiflorum	$\Delta g_s/\Delta c_a$	-1.202	[-5.808, 3.404]	14	EB
Dillenia biflora	$\Delta g_s/\Delta c_a$	-0.647	[-1.641, 0.347]	41	EB
Elattostachys falcata	$\Delta g_s/\Delta c_a$	0.557	[-0.574, 1.689]	44	EB
Gnetum gnemon	$\Delta g_s/\Delta c_a$	-0.134	[-0.804, 0.535]	41	EB
Amaroria soulameoides	$\Delta A_s/\Delta c_a$	0.019	[0.017, 0.02]	10,000	BiomeBGC*
Astronidium confertiflorum	$\Delta A_s/\Delta c_a$	0.026	[0.024, 0.027]	10,000	BiomeBGC*
Dillenia biflora	$\Delta A_s/\Delta c_a$	0.021	[0.017, 0.024]	10,000	BiomeBGC*
Elattostachys falcata	$\Delta A_s/\Delta c_a$	0.036	[0.036, 0.037]	10,000	BiomeBGC*
Gnetum gnemon	$\Delta A_s/\Delta c_a$	0.034	[0.033, 0.036]	10,000	BiomeBGC*
Amaroria soulameoides	$\Delta A_s/\Delta c_a$	-0.023	[-0.048, 0.002]	38	EB
Astronidium confertiflorum	$\Delta A_s/\Delta c_a$	0.024	[-0.012, 0.059]	14	EB
Dillenia biflora	$\Delta A_s/\Delta c_a$	0.014	[-0.003, 0.031]	41	EB
Elattostachys falcata	$\Delta A_s/\Delta c_a$	0.030	[0.013, 0.047]	44	EB
Gnetum gnemon	$\Delta A_s/\Delta c_a$	0.022	[0.013, 0.031]	41	EB
